# An injectable subunit vaccine containing Elongation Factor Tu and Heat Shock Protein 70 partially protects American bison from *Mycoplasma bovis* infection

**DOI:** 10.3389/fvets.2024.1408861

**Published:** 2024-06-26

**Authors:** Bryan S. Kaplan, Rohana P. Dassanayake, Robert E. Briggs, Carly R. Kanipe, Paola M. Boggiatto, Lauren S. Crawford, Steven C. Olsen, Harish Menghwar, Eduardo Casas, Fred M. Tatum

**Affiliations:** ^1^Ruminant Diseases and Immunology Research Unit, National Animal Disease Center, Agricultural Research Service, United States Department of Agriculture, Ames, IA, United States; ^2^Infectious Bacterial Diseases Research Unit, National Animal Disease Center, Agricultural Research Service, United States Department of Agriculture, Ames, IA, United States

**Keywords:** *Mycoplasma bovis*, bison, vaccine, subunit, EFTu, Hsp70

## Abstract

*Mycoplasma bovis* (*M. bovis*) is the etiologic agent of high mortality epizootics of chronic respiratory disease in American bison (*Bison bison*). Despite the severity of the disease, no efficacious commercial vaccines have been licensed for the prevention of *M. bovis* infection in bison. Elongation factor thermal unstable (EFTu) and Heat Shock Protein 70 (Hsp70, *DnaK*) are highly conserved, constitutively expressed proteins that have previously been shown to provide protection against *M. bovis* infection in cattle. To assess the suitability of EFTu and Hsp70 as vaccine antigens in bison, the immune response to and protection conferred by an injectable, adjuvanted subunit vaccine comprised of recombinantly expressed EFTu and Hsp70 was evaluated. Vaccinates developed robust antibody and cellular immune responses against both EFTu and Hsp70 antigens. To assess vaccine efficacy, unvaccinated control and vaccinated bison were experimentally challenged with bovine herpes virus-1 (BHV-1) 4 days prior to intranasal infection with *M. bovis*. Vaccinated bison displayed reductions in joint infection, lung bacterial loads, and lung lesions compared to unvaccinated controls. Together, these results showed that this subunit vaccine reduced clinical disease and bacterial dissemination from the lungs in *M. bovis* challenged bison and support the further development of protein subunit vaccines against *M. bovis* for use in bison.

## 1 Introduction

*Mycoplasma bovis* (*M. bovis*) is a small, wall-less bacteria of the class *Mollicutes,* a group of organisms harboring some of the smallest known, characterized genomes (1). *M. bovis* is an economically important pathogen of cattle that was first recognized as the etiologic agent of mastitis in dairy cows in 1961 (2). Now recognized as an endemic pathogen in most countries worldwide (3), *M. bovis,* in addition to being a major cause of mastitis, can cause chronic pneumonia and polyarthritis syndrome (CPPS) and is often associated with the polymicrobial bovine respiratory disease complex (BRD), with calves and young cattle being the primary demographic affected (4, 5). It is estimated that the economic impact associated with BRD in feedlot cattle is up to $800 million to $900 million annually in the United States (US), with costs attributed to lack of performance, veterinary care, and loss of animals (6).

*M. bovis* emerged as a significant pathogen of American bison (*Bison bison*) in 1999 (7) and has become a persistent threat to the health of ranched bison in the US and Canada. In bison, *M. bovis* is a primary respiratory pathogen causing high morbidity and mortality epizootics of pneumonia and polyarthritis with case fatality rates reaching 45% (8–10). A serological survey conducted in Canada found 79% of surveyed herds contained seropositive animals with up to 41% of animals being seropositive in herds (7), demonstrating the large number of animals affected during *M. bovis* outbreaks. Clinical disease in bison is described as pneumonia or pharyngitis with polyarthritis and involvement of other organs and tissues including laryngitis, pleuritis, pericarditis, and mastitis (9, 11, 12). The clinical signs of *M. bovis* infection in bison, which are often masked and difficult to identify, include dyspnea, coughing, sluggishness, swollen joints, lameness, and loss of body condition (8, 10). The primary affected demographics include yearlings and cows greater than 3 years of age (10), though calves have also been determined to be an affected population (13). Control and prevention of *M. bovis* infection is difficult due to the long incubation period and presence of chronic carriers that can confound efforts to control the disease through management practices.

Despite the large burden of disease, there are few commercially licensed vaccines for use in cattle and none for use in bison. Bacterin-based vaccines are currently available for use in cattle in the US though in field studies these vaccines failed to protect calves from clinical disease and shedding (4, 14, 15). More recently, a live-attenuated vaccine has been approved in the US though, as of this writing, there is no data available on its performance in the field. A major challenge for bacterin and live-attenuated vaccines stems from the antigenic diversity conferred through the variable surface proteins (VSP) (16–18). The antigenic diversity generated through VSP phase-variation presents a challenge that requires constant reformulation of vaccines and may not encompass the global antigenic diversity present for *M. bovis*. Alternatively, employing a subunit-based vaccine approach to this problem offers the distinct advantage of incorporating highly conserved *M. bovis* proteins selected to elicit broadly cross-protective immunity against disparate *M. bovis* strains. Moonlighting proteins are typically highly conserved enzymes or chaperones with more than one biological function that can contribute to bacterial virulence, thus making them attractive vaccine targets (19, 20). A recent study (21) has shown that elongation factor thermal unstable (EFTu) and Heat Shock Protein 70 (Hsp70) proteins of *Mycoplasma ovipneumoniae* are abundantly expressed, membrane-associated proteins that induced antibody and cellular responses in mice. Further, a modified-live *Mannheimia haemolytica* expressing truncated *M. bovis* EFTu and Hsp70 was effective in reducing lung bacterial loads and lung pathology following experimental challenge in calves (22), though these observations were not as pronounced when evaluated in bison.

The purpose of this study was to evaluate the protection conferred by an injectable adjuvanted subunit vaccine containing recombinantly expressed EFTu and Hsp70 peptides against intranasal *M. bovis* infection in North American bison. Results presented herein demonstrate that two doses of the experimental subunit vaccine were sufficient to induce antigen specific antibody and T cell responses. Following challenge with *M. bovis*, vaccination was associated with reduced lung pathology, bacterial loads, and dissemination of *M. bovis* to the joints. Together these findings support the continued development of EFTu and Hsp70 containing subunit vaccines for use in bison and cattle.

## 2 Materials and methods

### 2.1 Animals and housing

All experimental procedures were evaluated and approved by the National Animal Disease Center Animal Care and Use Committee (protocol # ARS-22-1035, approved 08/26/2022) prior to initiation of the study. Nine bison, aged 1–5 years, were randomly assigned to the vaccinate group (*n* = 5) or unvaccinated control group (*n* = 4). Both male and female bison were represented in each group. Table 1 details the demographics of animals in each study group. Prior to the study, all animals tested negative for *M. bovis* and bovine herpes virus-1 (BHV-1). *M. bovis* exposure was assessed via serology, as assessed by the BioX Monoscreen K302 *Mycoplasma bovis* ELISA Kit (BioX Diagnostics S.A., Rochefort, Belgium) according to manufacturer’s instruction, and bacterial isolation from nasal swabs. Bovine herpes virus-1 (BHV-1) infection status was assessed via nasal swab virus isolation. Detailed methods for bacterial and viral isolation are described in section 2.6. For the duration of the study, bison were housed individually in pens inside an Agricultural Biosafety level 3 containment facility as this is the only containment facility equipped for bison husbandry. A timeline of the experiment including milestones and samples collections is detailed in Table 2. At study end, bison were humanely euthanized by intravenous injection of sodium pentobarbitol (Ft. Dodge Labs, Fort Dodge, IA).

**Table 1:** Demographics of study groups.

Group	Animal ID	Age (years)	Sex
Vaccinate	496	5	Female
	507	5	Female
	510	3	Male
	513	3	Female
	516	1	Male
Control	503	5	Female
	511	3	Male
	512	3	Male
	515	1	Female

**Table 2:** Timeline of study evaluating the efficacy of a subunit vaccine against *Mycoplasma (M.) bovis*.

Day post-vaccination (DPV)	Day post-infection (DPI)	Event	Samples collected
0	−36	First vaccine dose administered^a^	Nasal swabs, whole-blood^b^, serum
21	−15	Second vaccine dose administered^a^	Nasal swabs, whole-blood^b^, serum
36	0	Intranasal inoculation with BHV1	Nasal swabs, whole-blood^c^, serum
38	2	Asses BHV-1 infection status	Nasal swabs
40	4	Intranasal inoculation with *M. bovis* cocktail	Nasal swabs, whole-blood^c^, serum
47	11	Assess *M. bovis* infection	Nasal swabs, whole-blood^c^, serum
51	15	End of study (511)	Nasal swabs, tissue samples, whole-blood^d^, serum
58	22	End of study (503)	Nasal swabs, tissue samples, whole-blood^d^, serum
63	27	End of study (512, 515)	Nasal swabs, tissue samples, whole-blood^d^, serum
64	28	End of study (507, 510, 516)	Nasal swabs, tissue samples, whole-blood^d^, serum
65	29	End of study (496, 513)	Nasal swabs, tissue samples, whole-blood^d^, serum

a Subcutaneous administration to the lateral cervical area.

b Whole-blood collected in ACD buffer.

c Whole-blood collected in ACD buffer and EDTA.

d Whole-blood collected in EDTA.

### 2.2 Subunit vaccine formulation

Recombinant EFTu and Hsp70 were produced for use as vaccine antigen as previously described (22). The EFTu and Hsp70 fragments were amplified by PCR and inserted into the pSumo system (Thermo Fisher, Waltham, MA) then transformed into BL21 (DE3) *Escherichia coli*. Recombinant Hsp70 protein was expressed and purified using a Ni-NTA column according to manufacturer’s direction and EFTu in inclusion body was solubilized in B-PER reagent (Supplementary Figure S1) (Thermo Fisher, Waltham, MA). One hundred micrograms of each protein was diluted in Dulbecco’s phosphate buffered saline (DPBS) and combined with the oil-in-water adjuvant Emulsigen-D (MVP Adjuvants, Omaha, NE) to a final concentration of 30% (v/v) in a final volume of 2 mL. Two doses of vaccine were administered subcutaneously in the lateral cervical area, the first (prime) on 0 days post-vaccination (DPV) and the second (boost) on 21 DPV.

### 2.3 Serology and complete blood counts

Bison blood was collected by jugular venipuncture into SST vacutainer tubes (BD, Franklin Lakes, NJ). Serum was separated via centrifugation at 1,200 RPM for 15 min at room temperature (RT), aliquoted in 2 mL cryovials, then stored at −80°C until use. The BioX Monoscreen K305 *M. bovis* ELISA kit (Bio-X Diagnostics, Jemelle, Belgium) was used to assess the presence of *M. bovis* specific antibodies in the blood of bison, 1 replicate per sample per timepoint, prior to study initiation and following experimental infection.

To assess vaccine antigen specific antibodies, serum samples were assayed using an in-direct ELISA as previously described (22). Immulon HB2 plates (Thermo Fisher) were coated with 1 μg/well recombinant Hsp70 and EFTu in a volume of 200ul/well of sodium carbonate-bicarbonate coating buffer (Sigma-Aldrich, St. Louis, MO). Coated plates were sealed and incubated overnight at 4°C prior to use. Plates were washed once and blocked using blocking buffer comprised of Tris buffered saline with 0.05% Tween 20, pH 8.0, 0.5% fish gelatin (Sigma-Aldrich), and 1% horse serum for 1 h at RT. Bison serum was diluted 1:50 in blocking buffer, added to coated wells, and incubated for 2 h at RT and then washed 3 times with blocking buffer. Bound antibodies were detected using Protein G conjugated with horse radish peroxidase (Sigma-Aldrich) diluted 1:1000 in Tris buffered saline and incubated for 1 h at RT. Plates were then washed 3 times with blocking buffer prior to detection with AP SigmaFast p-nitrophenyl tabs (Sigma-Aldrich). The optical density of assay plates was assessed using a Molecular Devices SpectraMax 250 plate reader (San Jose, CA) at a wavelength of 450nm. Normalized optical densities for all samples was calculated by subtracting the absorbance readings of the uncoated wells from that of the coated wells.

For complete blood counts (CBC), blood was collected in a vacutainer containing ethylenediaminetetraacetic acid (EDTA) (BD Biosciences). CBCs were determined using a Sysmex XN-V CBC analyzer (Sysmex America Inc., Lincolnshire, IL) with a bovine hematologic profile.

### 2.4 Peripheral blood mononuclear cell proliferation assays

Peripheral blood mononuclear cells (PBMC) were isolated from bison whole blood as previously described (23). Briefly, blood was collected by jugular venipuncture into 50 mL conical tubes containing acid citrate dextrose (ACD) buffer to a final ratio of 1:10 ACD-to-blood. Blood was then centrifuged at 1,200 × g for 30 min at RT. The buffy coat was removed and diluted 1:2 with DPBS and centrifuged again as described above (Thermo Fisher). The buffy coat was removed and layered onto a Ficoll gradient, 1.077 g/mL (Sigma-Aldrich) prior to centrifugation at 1,200 × g for 30 min at RT. PBMC were recovered, washed once in DPBS, and pelleted by centrifugation at 300 × g for 10 min at RT. PBMC live counts were determined using the Muse Cell Analyzer (MilliporeSigma, Burlington, MA) according to manufacturer’s recommendations. PBMC were then resuspended to a final concentration of 1 × 10^7^ cells/ml in complete RPMI 1640 (cRPMI) (Thermo Fisher) supplemented with 20% heat-inactivated fetal bovine serum, 100 U/mL penicillin, 100 μg/mL streptomycin, 2 nM glutamine, 1% sodium pyruvate, 1% non-essential amino acids, 1% essential amino acids (Sigma-Aldrich), 50 μM 2-mercaptoethanol (Sigma-Aldrich), and 1% HEPES buffer (Thermo Fisher).

Antigen specific PBMC proliferation was assessed via staining with CellTrace violet (Thermo Fisher), as previously described (24). Cells were cultured in 96-well flat bottom plates at a concentration of 1 × 10^6^ cells per well, 1 well per animal per timepoint, at 37°C, 5% CO_2_ for 7 days in media alone (no stimulation), in the presence of EFTu (2 μg/well) or Hsp70 (2 μg/well), or stimulated with Concanavalin A (0.5 μg/well) as a positive control. On day 7, PBMC were washed with DPBS and incubated with a fixable viability dye (Thermo Fisher) for 20 min at 4°C and then washed once in DPBS and once in FACS buffer. Proliferating γδ T cells were identified by staining first with mouse anti-bovine γδ T cell receptor antibody (IgG2b, TCR1-N24; Washington State University, Pullman, WA, United States) and subsequently with anti-mouse IgG2b BUV395 labeled secondary antibody (BD Biosciences, East Rutherford, NJ) each for 15 min at RT. CD4 and CD8 T cells were identified using a FITC labeled anti-bovine CD4 (IgG2a, CC8) and an APC-labeled anti-bovine CD8 (IgG2a, CC63) antibodies (Bio-Rad, Hercules, CA, United States), respectively, for 15 min at RT. Stained PBMCs were fixed using BD Cytofix/Cytoperm Fixation/Permeabilization kit (BD Biosciences) prior to being analyzed on a BD FACSymphony A5 flow cytometer (BD Biosciences) collecting 50,000 single cells per sample. FlowJo software (BD Biosciences) was utilized for data analysis and the data presented has been normalized to the number of proliferating cells observed on 0 DPV. The gating strategies used to define T cell subsets and proliferation are presented in Supplementary Figures S2, S3, respectively.

### 2.5 BHV-1 and *Mycoplasma bovis* inoculum

*Mycoplasma bovis* inoculum was prepared as previously described (25). Axenic cultures isolated from single colonies were cultured and expanded in PPLO broth (BD Diagnostic Systems, United States) supplemented with 10 g/L of yeast extract (BD Diagnostic Systems) and 20% horse serum (v/v) (Thermo Fisher). Each isolate was grown for 48 h under aerobic conditions at 37°C, 5% CO_2_ and then pelleted by centrifugation at 15,000 × g for 20 min prior to resuspension at a 1/100 volume of fresh PPLO broth. The concentrated bacteria suspension was passed through a 25-gauge needle. Aliquots were stored at −80°C until use. A cocktail of 5 isolates (Table 3) was used as the virulence properties of individual *M. bovis* isolates in bison is currently unknown. On the day of infection, *M. bovis* stocks were thawed and 4 × 10^10^ CFU/mL of each isolate was combined for a final inoculum concentration of 2 × 10^11^ CFU/mL that was then drawn into 5 mL syringes and stored on ice until use.

**Table 3:** Composition of *Mycoplasma bovis* inoculum.

Isolate	ST^a^	Geographic origin	Isolation year
NADC1	1	Montana, USA	2011
NADC15	1	North Dakota, USA	2007
NADC16	2	Manitoba, Canada	2012
NADC30	2	South Dakota, USA	2012
KRB5	47	Kansas, USA	2014

a MLST sequence type (ST).

BHV-1 Cooper strain was kindly provided by Melinda Jenkins-Moore at the USDA-APHIS National Veterinary Service Laboratory (Ames, IA, United States). Five milliliters (2.5 mL per nostril) of clarified cell-culture suspensions, at titer, was used for inoculum. Both BHV-1 and *M. bovis* inocula were administered intranasally using an Intranasal Mucosal Atomization Device (MAD Nasal, Teleflex, Morrisville, NC).

### 2.6 Isolation and quantitation of bacteria and virus from swabs and tissues

To assess BHV-1 infection status, nasal swabs (Puritan PurFlock Ultra, Puritan LLC, Guilford, ME) were collected and stored on ice until used for virus isolation. One milliliter DPBS was added and 500 μL was used to inoculate bovine nasal turbinate (Btu) cells in 12-well plates. Briefly, Btu cells were incubated at 37°C, 5% CO_2_ for 1 h, washed twice with minimum essential medium (MEM) and cultured in 1 mL MEM (Thermo Fisher) supplemented 10% fetal bovine serum, L-glutamine, and antibiotic, antimycotic solution (Thermo Fisher) and incubated for 72 h at 37°C, 5% CO_2_. Btu cells were harvested by freezing the cell culture plates at −80°C. Cell culture supernatant was passaged onto fresh Btu cells, as described above, for a total of 2 passages. Samples that resulted in visible cytopathic effect were considered positive for BHV-1.

For *M. bovis* isolation and quantification, nasal and tissue swabs were collected using BD universal swabs for *Mycoplasma* specimens (BD Biosciences) and stored in 150 μL selective PPLO broth (PPLO broth supplemented with 0.05% thallium acetate and 500 IU/mL penicillin G) to prevent desiccation and stored on ice for transport to the lab. Swabs were then used to inoculate 3 mL of selective PPLO broth, which was cultured for 48 h at 37°C, 5% CO_2_. Following incubation, 5 μL of culture media was used as a template for PCR targeting the uvrC gene of *M. bovis* using previously described methods (26).

For bacterial quantification from tissue, Earle’s balanced salt solution was added to the tissue to a final ratio 10:1 (volume/weight). Tissue sections (1 to 3 g) from the left cranial lung, left middle lung, accessory lung, right cranial lung, and right middle lung were collected and homogenized using a Tissumizer homogenizer (Tekmar Company, Cincinnati, OH). Ten-fold dilutions of lung homogenates in DPBS were spotted onto selective PPLO agar plates (PPLO agar, 0.05% thallium acetate and 500 IU/mL penicillin G) in triplicate. Following 72 h incubation at 37°C, 5% CO_2_, colonies were counted and used for calculation of CFU.

### 2.7 Gross pathology and histopathology

Lungs were extracted *in toto* for estimation of lesion volume as previously described (22). Lung lesion volumes were visually estimated for each lung lobe. A summation of scores for the 8 individual lobes was calculated and multiplied by an approximate percentage of each lung lobes contribution to air exchange. Approximate contribution of each lung lobe to air exchange is calculated as follows: left cranial, 4%; left middle, 6%; left caudal, 32%; accessory, 4%; right cranial, 6%; cranial half of right middle, 5%; caudal half of right middle, 7%; right caudal, 35%. Following examination of lungs for lesions and estimation of lung involvement, specimens displaying pathology were preferentially selected for histopathology. For each tissue, 1 to 3 g specimens were collected from abnormal areas, when present, in each of the following: left cranial lung, left middle lung, accessory lung, right cranial lung, and right middle lung as well as the palatine tonsil, retropharyngeal lymph nodes, and tympanic bullae. Samples were fixed in 10% neutral buffered formalin for 24 h then transferred to 70% ethanol, processed by routine paraffin embedding techniques, and cut in 4 μm sections prior to staining with hematoxylin and eosin and microscopic analysis using a Nikon Eclipse Ci with a Nikon DS-Ri2 camera (Nikon United States, Melville, NY, United States).

### 2.8 Statistical analysis

Statistical analysis was performed using GraphPad Prism version 9.5.1 (GraphPad Software, Boston, MA). Two-Way ANOVA with Tukey’s multiple comparisons test was used to evaluate statistical differences between groups. Group differences were adjudicated to be statistically significant when *p* ≤ 0.05.

## 3 Results

### 3.1 Seroconversion and T cell responses to *Mycoplasma bovis* vaccine antigens

**Figure 1:**
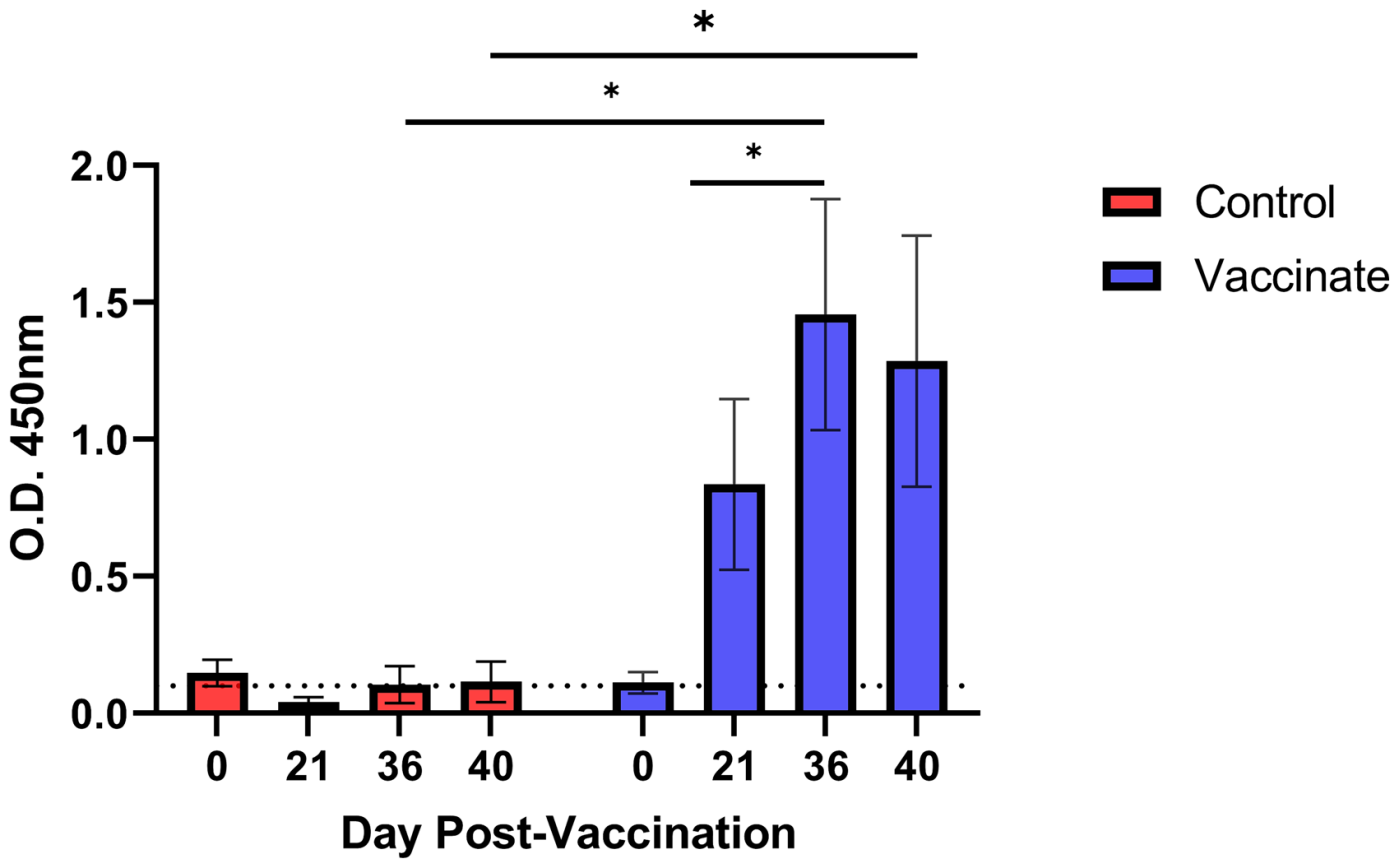
Antibody response to *Mycoplasma bovis* EFTu and Hsp70 antigens following vaccination in bison. Five bison were vaccinated with an experimental subunit vaccine incorporating purified EFTu and Hsp70 proteins on 0 and 21 days post vaccination (DPV). Serum antibody titers were assessed on 0, 21, 36, and 40 DPV by indirect ELISA. Presented are means ± SEM. The dotted line indicates the level of background absorbance at an optical density (O.D. of 450 nm) of 0.1. ^*^p ≤ 0.05.

Prior to initiation of the study, all animals in both unvaccinated control and vaccinate groups were seronegative for the vaccine antigens, EFTu and Hsp70 (Figure 1), in addition to being seronegative for *M. bovis* via commercial ELISA. Vaccinated bison produced vaccine specific antibodies following the first dose of vaccine as assessed on 21 DPV. A significant increase in vaccine specific antibody titers was observed 15 days following administration of the second vaccine dose as assessed on 36 DPV, with antibody titers being maintained until the day of infection with BHV-1 on 36 DPV and *M. bovis* on 40 DPV. Vaccine specific antibody titers were not observed in samples collected from unvaccinated control animals.

**Figure 2:**
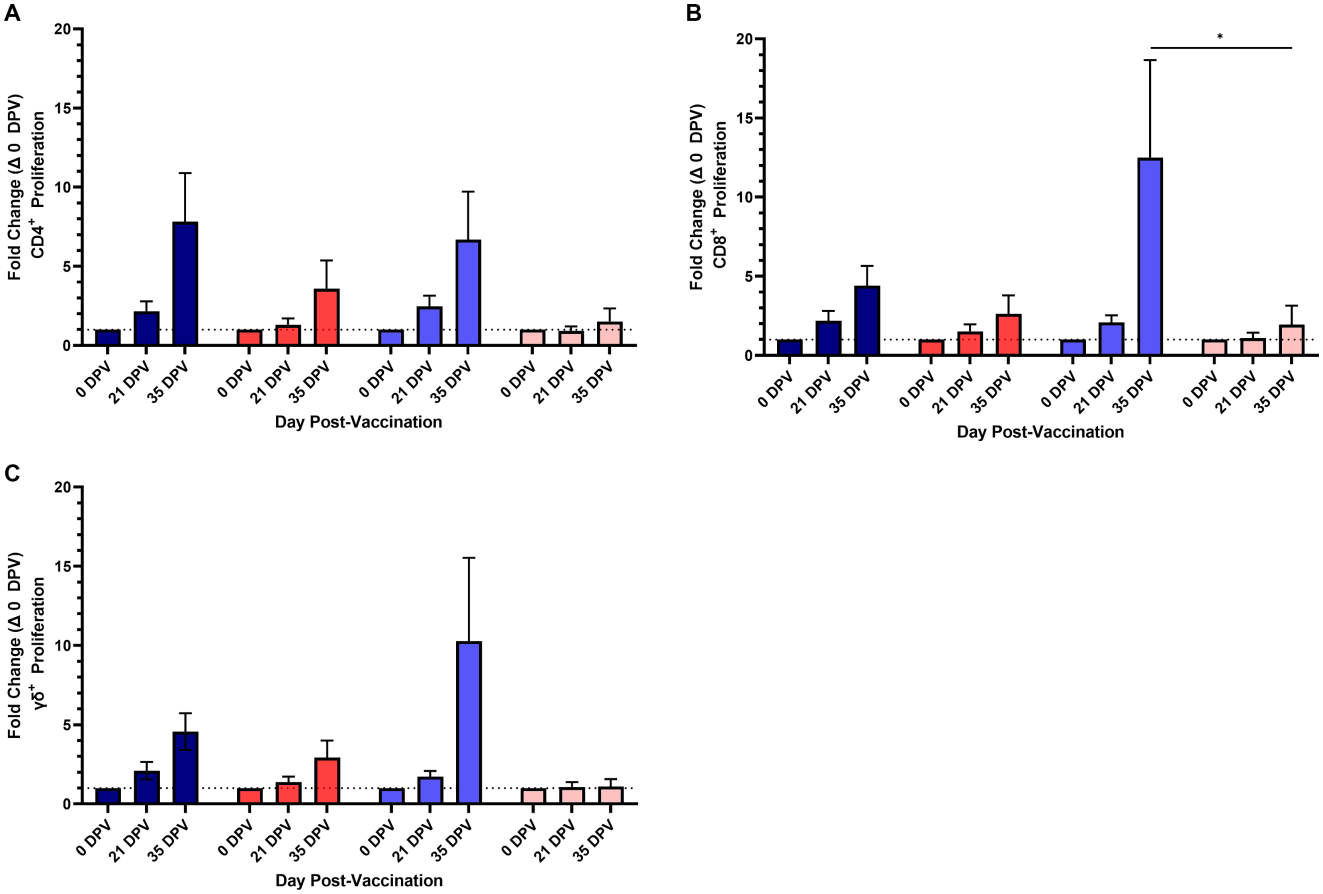
Proliferation of T cell subsets to *Mycoplasma bovis* EFTu and Hsp70 vaccine antigens in vaccinated and unvaccinated control bison. A time-course showing the fold-change of proliferating CD4 **(A)**, CD8 **(B)**, and γδ **(C)** T cell subsets following stimulation shows two vaccine doses greatly increase the number of antigen specific cells. The number of proliferating cells from vaccinates responding to Hsp70 (dark blue) and EFTu (light blue) are increased compared to cells from unvaccinated control bison treated with Hsp70 (red) and EFTu (pink). Presented are means ± SEM. The dotted line indicates baseline proliferative levels on 0 DPV. ^*^p ≤ 0.05.

Antigen specific cellular responses were assessed using PBMC isolated from vaccinates and unvaccinated control animals on 0, 21, and 36 DPV. PBMC were stimulated with one of the two vaccine antigens (either EFTu or Hsp70) and the frequency of proliferating CD4, CD8, and γδ T cells was determined. Two doses of vaccine were required to induce measurable proliferation in T cell subsets. The population of CD 4 T cells responding to *in vitro* antigen stimulation peaked on 36 DPV with 7.82 and 6.69% increases to the means of cells proliferating in response to Hsp70 and EFTu, respectively (Figure 2A). In contrast to the similar proliferative responses of CD4 T cells to both antigens, CD8 and γδ T cells displayed increased proliferation when stimulated with EFTu compared to Hsp70. The mean frequency of proliferating CD8 T cells on 36 DPV increased to 12.5% when stimulated with EFTu compared to 4.4% with Hsp70 (Figure 2B). The proliferative responses of γδ T cells, similar to that observed in the CD8 T cell subset, showed a greater response observed following *in vitro* stimulation with EFTu (10.28%) and Hsp70 (4.57%) (Figure 2C). Together, these data demonstrate that an adjuvanted subunit vaccine can induce antibodies and T cell proliferative responses targeting purified *M. bovis* EFTu and Hsp70 proteins in bison.

### 3.2 Vaccine reduced *Mycoplasma bovis* dissemination and lung titers in bison

Bison in both groups were intranasally infected with BHV-1 on 36 DPV (0 DPI) followed by *M. bovis* on 40 DPV (4 DPI). Nasal swabs collected prior to the administration of each vaccine dose and challenge were negative BHV-1 as determined by virus isolation on bovine turbinate cells. Nasal swab samples collected from all animals on 2 DPI were positive by virus isolation confirming that all animals were infected with BHV-1 (data not shown). Nasal swabs collected on 11 DPI (47 DPV) were assessed for *M. bovis* by PCR and culture. Nasal swab samples from the four unvaccinated controls and five vaccinates were positive for *M. bovis* via uvrC PCR while four samples (4/4) from unvaccinated bison and five (5/5) samples from vaccinates were positive by culture (Table 4). These data show successful intranasal infection of bison with both BHV-1 and *M. bovis*.

**Table 4:** Detection of *Mycoplasma bovis* in nasal swab samples from experimentally infected bison.

	0 DPV		21 DPV		36 DPV		47 DPV	
	PCR	Culture	PCR	Culture	PCR	Culture	PCR	Culture
Control	0 (0)^a^	0 (0)	0 (0)	0 (0)	0 (0)	0 (0)	4 (1)	4 (1)
Vaccinate	0 (0)	0 (0)	0 (0)	0 (0)	0 (0)	0 (0)	5 (1)	5 (1)

a Number of animals positive for *Mycoplasma bovis* (frequency of positive swabs).

**Figure 3:**
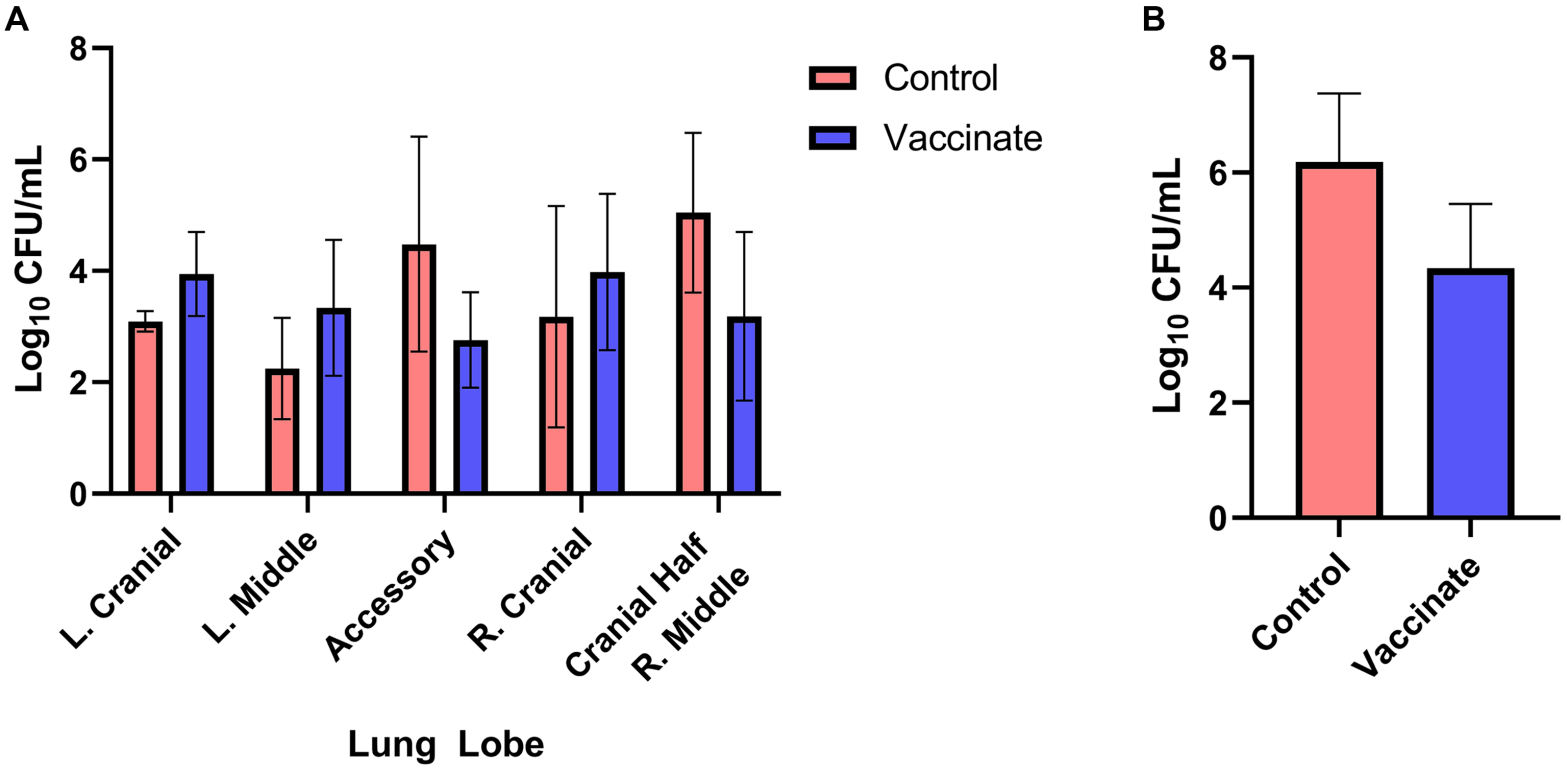
*Mycoplasma bovis* titers in the lungs of vaccinated and unvaccinated bison. Sections of the left cranial, left middle, accessory, right cranial, and the cranial half of the right middle lung lobes were collected, homogenized, and serial dilutions plated on selective PPLO agar plates for quantitation of *Mycoplasma bovis*. Bacteria lung titers (CFU/mL) from unvaccinated controls (red) and vaccinates (blue) displayed no pattern between individual lung lobes **(A)** though the average bacterial lung was lower in vaccinates compared to controls **(B)**. Presented are means ± SEM.

On 27–29 DPI (63–65 DPV) bison were euthanized to assess bacterial loads and tissue pathology, with the exceptions of bison numbers 511 and 503. Each of these animals was euthanized prior to the scheduled study end study end on 15 and 22 DPI, respectively, as each animal displayed lameness, tachypnea, labored breathing, and inappetence. For all animals, sections of select lung lobes were collected and homogenized to quantify bacterial titers and determine if vaccination reduced bacterial load in the lungs. Assessment of bacterial titers in individual lung lobes did not reveal a distinct pattern of colonization (Figure 3A), yet averages for the total lung found vaccinated bison to have lower *M. bovis* titers (4.34 CFU/mL) compared to unvaccinated controls (6.19 CFU/mL) (Figure 3B).

At necropsy, additional swabs were collected from the nasal cavity, trachea, middle ear, and synovium to assess colonization. These results are summarized in Table 5. The nasal swabs collected from all bison in both groups tested positive for *M. bovis* by both PCR and culture. Identical results were obtained from tracheal and middle ear swabs however differences were observed from synovial swabs. All joint swabs collected from unvaccinated control bison tested positive for *M. bovis* via PCR and *M. bovis* was cultured from three of four. In contrast, two of five synovial swabs from vaccinated animals were PCR positive and *M. bovis* was cultured from just one vaccinated animal. Together, these results indicate that vaccination with EFTu and Hsp70 may provide sufficient immunity to control *M. bovis* infection in the lungs and reduce colonization of sites outside of the respiratory tract.

**Table 5:** Detection of *Mycoplasma bovis* in tissue swabs of experimentally infected bison at necropsy.

	Nasal cavity		Trachea		Middle ear		Joint	
	PCR	Culture	PCR	Culture	PCR	Culture	PCR	Culture
Control	4 (1)^a^	4 (1)	4 (1)	4 (1)	4 (1)	4 (1)	4 (1)	3 (0.75)
Vaccinate	5 (1)	5 (1)	5 (1)	5 (1)	5 (1)	5 (1)	2 (0.4)	1 (0.2)

a Number of animals positive for *Mycoplasma bovis* (frequency of positive swabs).

### 3.3 Vaccinated bison displayed reduced lung pathology following experimental challenge with *Mycoplasma bovis*

**Figure 4:**
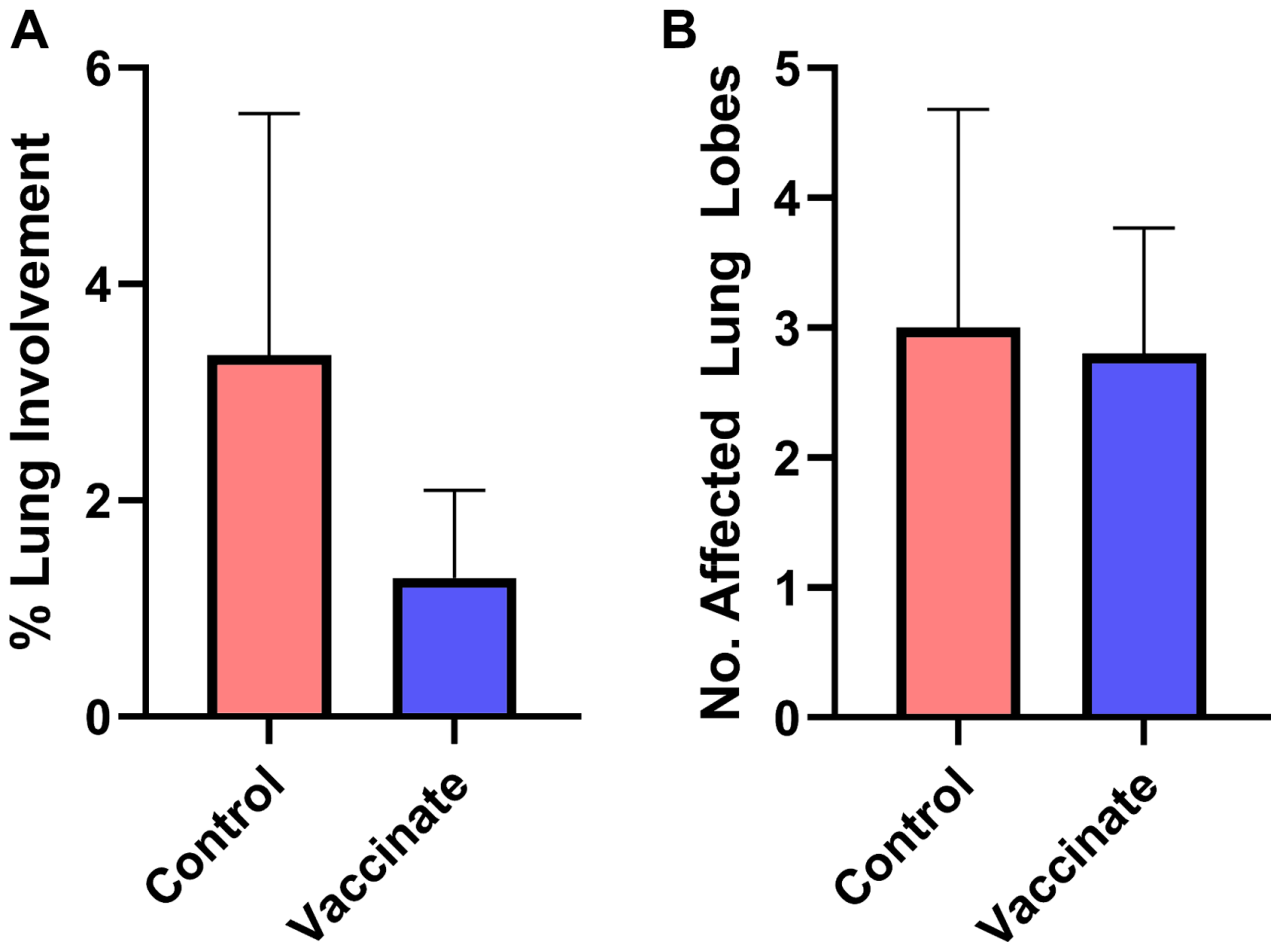
Vaccination reduces the lung pathology score following *Mycoplasma bovis* challenge in bison. The lungs of vaccinates (blue) and unvaccinated controls (red) were examined for the formation of lesions and scored based on the percentage of area affected. The lungs of vaccinated bison had reduced lung involvement compared to unvaccinated controls **(A)** despite no difference in the number of lung lobes displaying pathology **(B)**. Presented are means ± SEM.

The lungs of bison were examined for the presence of gross pathology and histopathology. The lungs of vaccinated bison showed a reduction in lung pathology scores compared to those of unvaccinated control animals (Figure 4A) though there was no difference in the number of affected lung lobes between the groups (Figure 4B). The size of the lung lesions in vaccinated bison were reduced (average diameter: 0.2 cm, range: 0.08–0.4 cm) compared to those observed in the lungs of unvaccinated controls (average diameter: 5.46 cm, range: 0.1–15 cm). The lungs of unvaccinated bison were characterized as having multifocal to coalescing grey to dark red lesions of mixed size with firmness ranging from normal to nearly hepatized in the cranial, middle, and caudal lobes. Interestingly, white to pale yellow caseonecrotic material was observed on cut section in the lungs of multiple animals as well as the palatine tonsils of bison 503, euthanized prior to study end due to severe clinical signs. The lungs of vaccinated bison were primarily normal, with the lungs of three bison showing small, well-demarcated lesions and one displaying a small abscess with white exudate.

**Figure 5:**
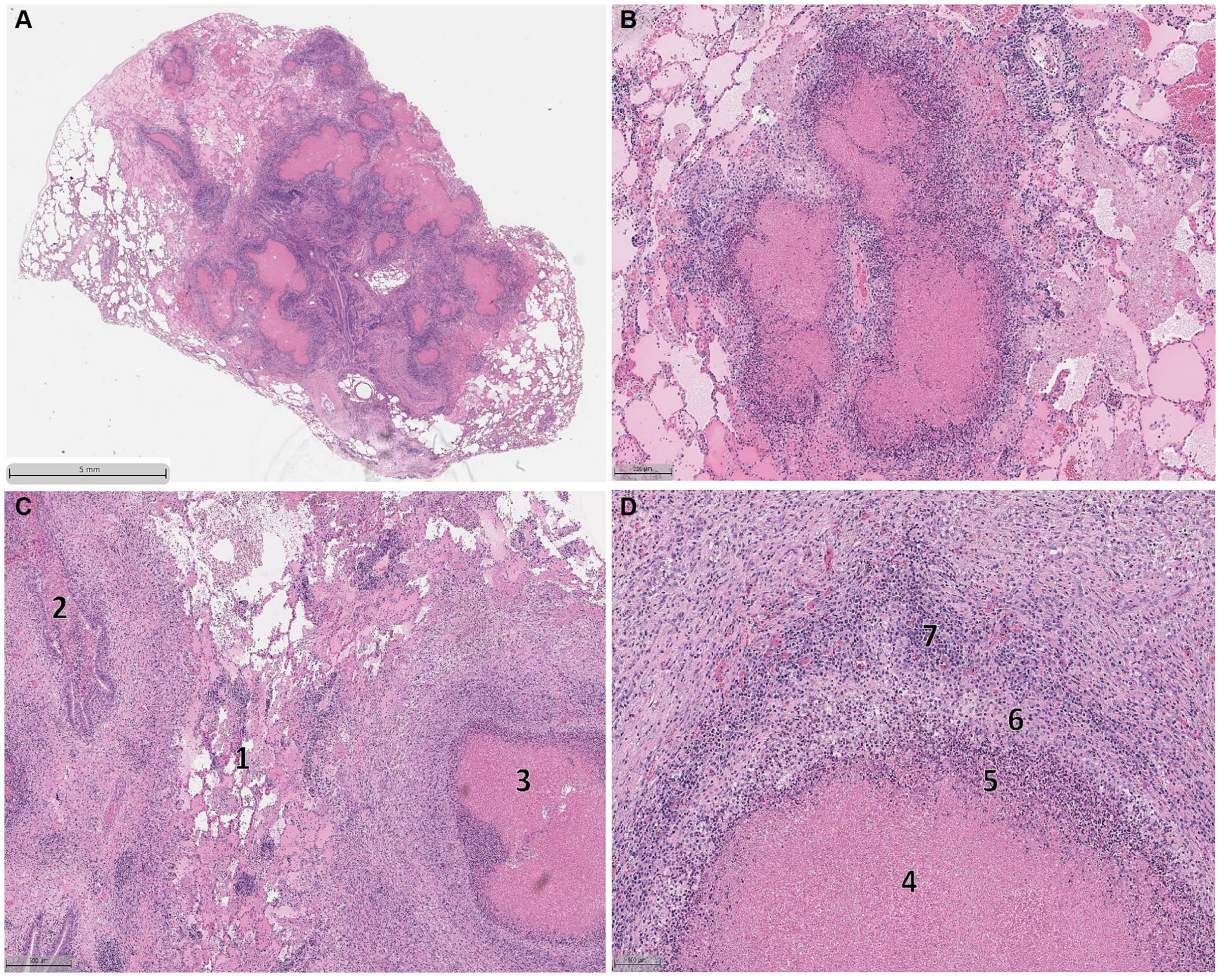
Histologic lesions observed in the lungs of bovine herpes virus-1, *Mycoplasma bovis* co-infected bison. Lung sections demonstrating variable, progressive disease severity including multifocal areas of hypereosinophilic caseonecrosis originating in the bronchioles and alveoli containing seroproteinaceous fluid **(A,B)**. The peripheral areas of the lungs are less affected. Different stages of lesion formation could be simultaneously observed **(C)** with early stages of lesion formation characterized by alveolar congestion with seroproteinaceous fluid and fibrin (1) and necrotizing exudate within the bronchioles (2) with established lesions displaying more structural organization (3). A section of an established lesion **(D)** displaying a concentric pattern of necrosis and inflammation characterized by hypereosinophilic caseonecrotic core (4) surrounded by a layer of neutrophils (5), macrophages (6), and ultimately lymphocytes and plasma cells (7).

Histological abnormalities were observed in the lungs of bison from both groups. Specimens collected from these lungs demonstrated variable, progressive disease severity. Consistent throughout the lesions were multifocal areas of hypereosinophilic caseonecrosis, originating within the bronchioles and seroproteinaceous fluid present in the alveoli, while peripheral lung parenchyma was less affected (Figures 5A,B). In some instances, necrotizing exudate was found within the bronchioles (Figure 5C). Lung lesions demonstrated a concentric pattern of necrosis and inflammation typical of *M. bovis,* characterized by a central area of hypereosinophilic caseonecrosis surrounded by neutrophils and macrophages followed by areas of lymphocytes and plasma cells (Figures 5C,D).

### 3.4 Elevated blood neutrophil and monocyte counts precedes severe clinical disease in *Mycoplasma bovis* infected bison

**Figure 6:**
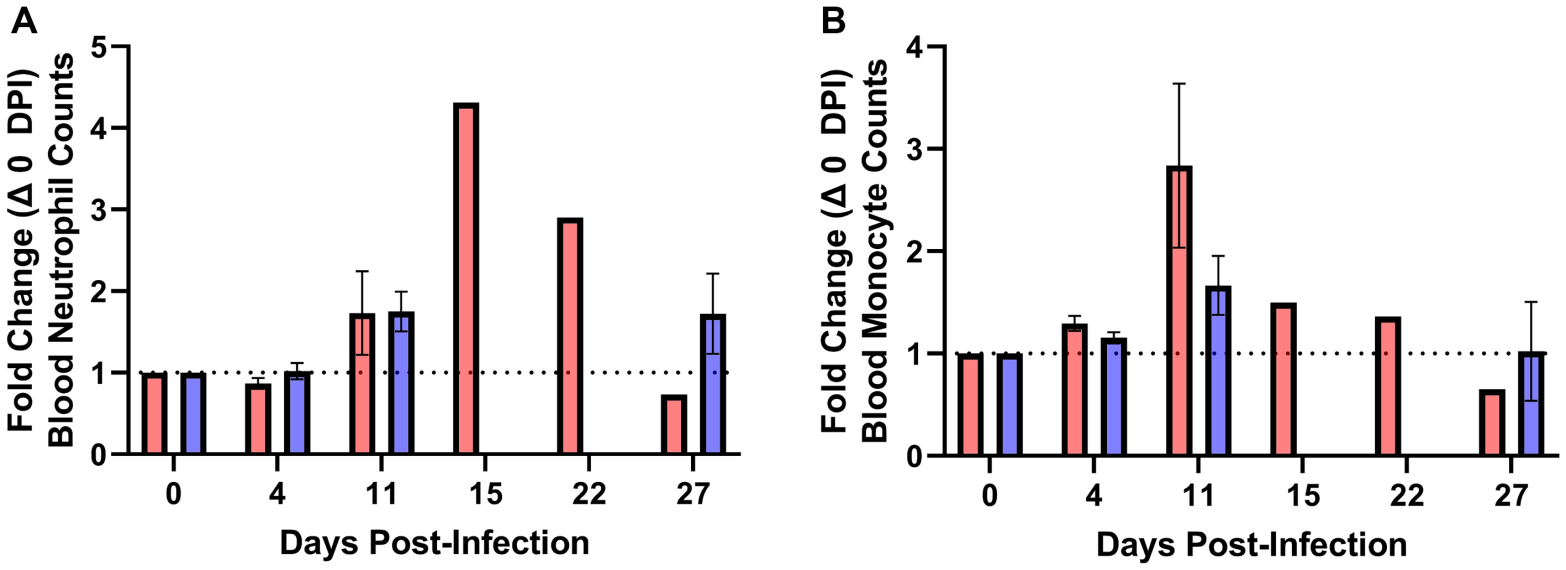
Elevated blood neutrophil and monocyte counts precede severe *Mycoplasma bovis* disease in bison. Complete blood counts were used to quantify the number of neutrophils and monocytes over the duration of the experiment. Moribund bison had a sharp increase in the number of neutrophils relative to 0 DPV **(A)** prior to exhibiting clinical signs and reaching clinical endpoints on 15 and 22 DPI. An increase in blood monocytes relative to 0 DPV **(B)** was observed in unvaccinated animals beginning on 11 DPV that remained elevated in moribund control animals on 15 and 22 DPV. Presented are means ± SEM.

Blood was collected for complete blood counts (CBC) over the duration of the challenge phase of the study. Beginning approximately 1 week following challenge with *M. bovis* (11 DPI), increases in the proportions of neutrophils (Figure 6A) and monocytes (Figure 6B) were observed for both vaccinates and unvaccinated control animals. On 15 DPI, bison 511 was euthanized for exhibiting severe clinical signs and elevated neutrophil and monocyte counts were observed in peripheral blood samples. Elevated neutrophil and monocyte counts were also obtained for bison 503 that was euthanized on 22 DPI after exhibiting severe clinical signs. In contrast, the proportion of neutrophils and monocytes in the blood of vaccinates remained relatively consistent over the course of the experiment with the exceptions of 11 DPV (1.75-fold neutrophils, 2.83-fold monocytes) and 27 DPV (1.72-fold neutrophils, 0.65-fold monocytes).

## 4 Discussion

*M. bovis* continues to pose a significant threat to American bison. Despite this, there are no vaccines currently licensed for use in this species. The data detailed above represent a promising advancement towards the development of an effective vaccine against *M. bovis* for use in bison. Specifically, the reduction of clinical signs (inappetence, lameness, tachypnea, labored breathing) reduced lung pathology, and decreased lung bacterial loads and dissemination to the joints suggest that EFTu and Hsp70 are protective antigens in bison when administered in an injectable, adjuvanted vaccine. Additionally, this is the first reporting of a BHV-1, *M. bovis* coinfection model in bison despite *M. bovis* being considered a primary pathogen in bison. In laboratory studies, co-infections of cattle with BHV-1 (27), Influenza D Virus (28), or *Mannheimia haemolytica* (29) and *M. bovis* have all been shown to increase clinical disease compared to single *M. bovis* infections as have intratracheal infection (30) and aerosol exposure (31). A BHV-1, *M. bovis* co-infection was selected for this study as it is a minimally invasive procedure and due to previous experience with this model (22).

Coinfection of bison with BHV-1 and *M. bovis* can induce severe clinical disease in bison. In this study, half of the unvaccinated control animals displayed inappetence, lameness, tachypnea, and labored breathing which are common clinical signs of *M. bovis* infection in bison (9) and cattle (4). Compared to a previous bison challenge study using intranasal infection of *M. bovis* alone (25), coinfection greatly increased the frequency and size of lung lesions in the unvaccinated control group though the histologic lesions observed in coinfected bison displayed similar pathological findings, namely purulent bronchopneumonia with lymphohistiocytic infiltrates and granuloma formation in the parenchyma. Additionally, the caseonecrosis noted in the lungs and tonsils of bison in this study was characteristic of the clinical presentation noted in naturally occurring *M. bovis* infection of bison (11, 12, 32) and experimentally infected cattle (27, 30). The elevated blood neutrophil counts observed in bison exhibiting severe disease is an interesting finding that has been previously shown to be associated with poor outcomes of pneumonia in humans and enhanced disease in mice with *Mycoplasma pneumoniae* (33, 34), though has never been reported during *M. bovis* infection. Together these findings suggest that BHV-1, *M. bovis* coinfection can recapitulate the pathology observed in naturally infected bison which is similar to that observed in cattle. Though *M. bovis* is a primary pathogen in bison, the coinfection model has utility for continued use in vaccine development and pathogenesis studies as it is currently the most effective method to recapitulate the tissue pathology observed in naturally infected bison with the benefit of a shorter incubation time as was observed with half of the control animals reaching clinical endpoints prior to the set study end. Despite this, further refinement of the model in warranted, specifically increasing the consistency and severity of clinical disease and pathology.

Protein subunit vaccines represent an attractive strategy for the prevention of *M. bovis* in bison and cattle as vaccine antigens could be combined with adjuvant to increase and shape the immune response as well as be included in a multivalent vaccine targeting bacterial and viral pathogens. In the past decade, significant progress has been made towards an effective protein subunit vaccine and several studies using reverse vaccinology have been successful in identifying conserved *M. bovis* proteins that are immunogenic in cattle. The *M. bovis* glyceraldehyde-3-phosphate dehydrogenase (GAPDH) protein was assessed as a potential vaccine antigen after it was discovered that calves produced antibodies against GAPDH following infection (35) though this protein did not provide protection to vaccinated calves in challenge studies (36). Subsequent studies utilizing a cocktail of 10 highly conserved *M. bovis* proteins examined the immunological effects of adjuvants and route of immunization and identified a mixture of adjuvants providing a more balanced IgG1/IgG2 antibody response following two vaccine doses administered intramuscularly (37, 38). A recent study found that although antisera from vaccinated calves was capable of fixing complement and killing *M. bovis in vitro,* complement-mediated killing was not sufficient to reduce bacterial loads and lung pathology (39), suggesting other, currently unknown mechanisms are responsible for mediating protection. EFTu has been identified as a protective antigen in *Burkholderia pseudomallei* (40), *Streptococcus pneumoniae* (41), and *Streptococcus suis* (42) suggesting EFTu is a suitable vaccine antigen for some bacterial species. Other approaches may also be beneficial in identifying correlates of protection and antigen selection including characterizing the role of antibodies in opsonization, phagocytosis, and inhibition of adhesion or other pathogenic functions.

The experimental vaccine evaluated herein induced measurable antigen specific proliferative responses across all three major T cell subpopulations. Surprisingly, the greatest change was observed in the CD8 and γδ T cell subsets. The adjuvant utilized in this study was Emulsigen-D that is known to induce strong T helper 1 (Th1) and antibody responses (43). *M. bovis* has been shown to invade bovine epithelial cells and PBMCs *in vitro* (44, 45). Th1 immunity is effective in combating intracellular pathogens and the induction of antigen specific CD8 T cells would potentially serve to clear potential reservoirs of bacterial replication in infected hosts. In bovines, γδ T cells comprise between 15 and 60% of the circulating lymphocytes and are noted for functional heterogeneity with subsets exhibiting an innate-like and regulatory phenotypes (46–48). Previous studies have shown γδ and CD8 T cells respond to *M. bovis* infection in cattle, though the functions of these cells during *M. bovis* infection is unclear (30, 49). Further immune exhaustion, as measured by increased expression of PD-1 in CD4 and CD8 T cells, has been observed during chronic *M. bovis* infections in cattle (50), though PD-1 expression by γδ T cells during *M. bovis* infection has not been reported. Studies examining the functions of bovine γδ T cells during *Mycobacterium bovis* infection, another chronic bacterial infection, show these cells play a fundamental role in shaping the immune response (51). The WC1.1^+^ and WC1.2^+^ γδ T cell subpopulations were both shown to proliferate in response to stimulation with *Mycobacterium bovis* antigens with the WC1.1^+^ cells producing IFN-γ (52). Though not shown for *Mycobacterium bovis* infection, WC1.2^+^ cells have been shown to produce IL-10 and have an immunosuppressive phenotype (46), suggesting a role for these cells in reducing immunopathology during chronic infection. Future studies evaluating γδ T cell phenotypes and subsets would be beneficial in identifying potential correlates of protection for *M. bovis* infection.

Currently there is no vaccine capable of preventing *M. bovis* infection in bison despite continued mass-mortality epizootics. The major findings from this study are two-fold. First, co-infection of American bison with BHV-1 followed by *M. bovis* induced disease in following intranasal challenge. The pathology observed in this challenge model recapitulated that observed in the field though to a lesser extent. Future studies evaluating different BHV-1 and *M. bovis* isolates, routes of infection, and increased study duration may be useful in enhancing the induced disease and refining the challenge model. Second, vaccination with EFTu and Hsp70 provided partial protection from observable clinical disease (inappetence, lameness, tachypnea, and labored breathing) following experimental *M. bovis* challenge despite the small sample size in both study groups. Altering vaccine composition using different adjuvants, increasing vaccine antigen content, and inclusion of additional antigens represent useful strategies to improve the vaccine evaluated herein. Increased knowledge of *M. bovis* pathogenic mechanisms and the host immune response will greatly facilitate the development of more protective vaccines for use in bison and cattle.

## Data availability statement

The original contributions presented in the study are included in the article/Supplementary material, further inquiries can be directed to the corresponding author.

## Ethics statement

The animal study was approved by USDA-ARS NADC Institutional Animal Care and Use Committee. The study was conducted in accordance with the local legislation and institutional requirements.

## Author contributions

BK: Conceptualization, Formal Analysis, Data curation, Investigation, Visualization, Writing – original draft, Writing – review & editing. RD: Conceptualization, Formal Analysis, Investigation, Writing – original draft, Writing – review & editing. RB: Writing – original draft, Writing – review & editing. CK: Formal analysis, Investigation, Visualization, Writing – original draft, Writing – review & editing. PB: Investigation, Writing – original draft, Writing – review & editing. LC: Investigation, Writing – original draft, Writing – review & editing. SO: Investigation, Writing – original draft, Writing – review & editing. HM: Investigation, Writing – original draft, Writing – review & editing. EC: Investigation, Writing – original draft, Writing – review & editing. FT: Investigation, Resources, Writing – original draft, Writing – review & editing.

## Supplementary material

Supplementary Figure S1 Purified recombinant *Mycoplasma bovis* Hsp70 and EFTu peptides used in this study.

Supplementary Figure S2 Gating strategy for flow cytometry analysis of bison lymphocytes. Representative dot plots show the gating strategy used to define lymphocytes, singlets, live cells, and major T cell subsets (CD4, CD8, γδ).

Supplementary Figure S3 Proliferation of bison T cell subsets. Representative histograms showing the CellTrace Violet staining of CD4, CD8, and γδ T cells following culture in media only, *Mycoplasma bovis* Hsp70 and EFTu, and ConA.
