# Supplementary figures and images for: An injectable subunit vaccine containing Elongation Factor Tu and Heat Shock Protein 70 partially protects American bison from Mycoplasma bovis infection
Source: Front Vet Sci. 2024 Jun 26;11:1408861. doi: 10.3389/fvets.2024.1408861 (PMC11234848; doi:10.3389/fvets.2024.1408861)

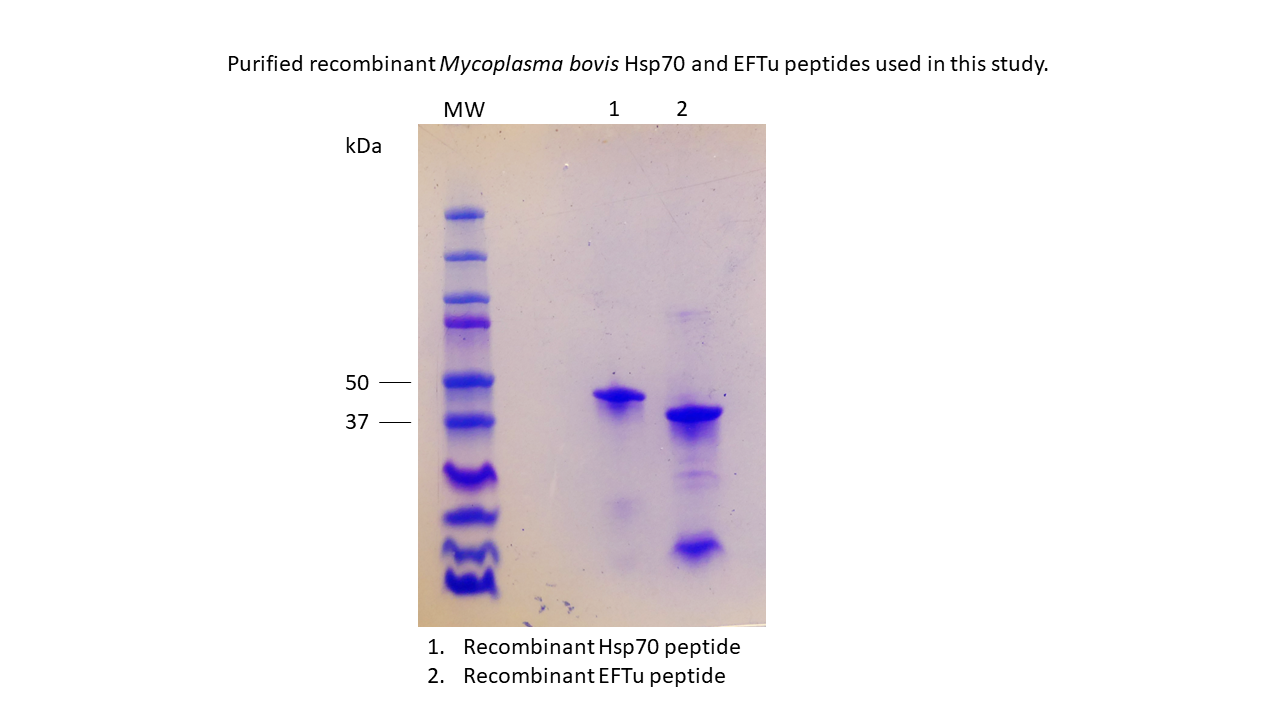

Supplement: Supplementary Figure S1 — Purified recombinant Mycoplasma bovis Hsp70 and EFTu peptides used in this study. [file Image_1.TIF]

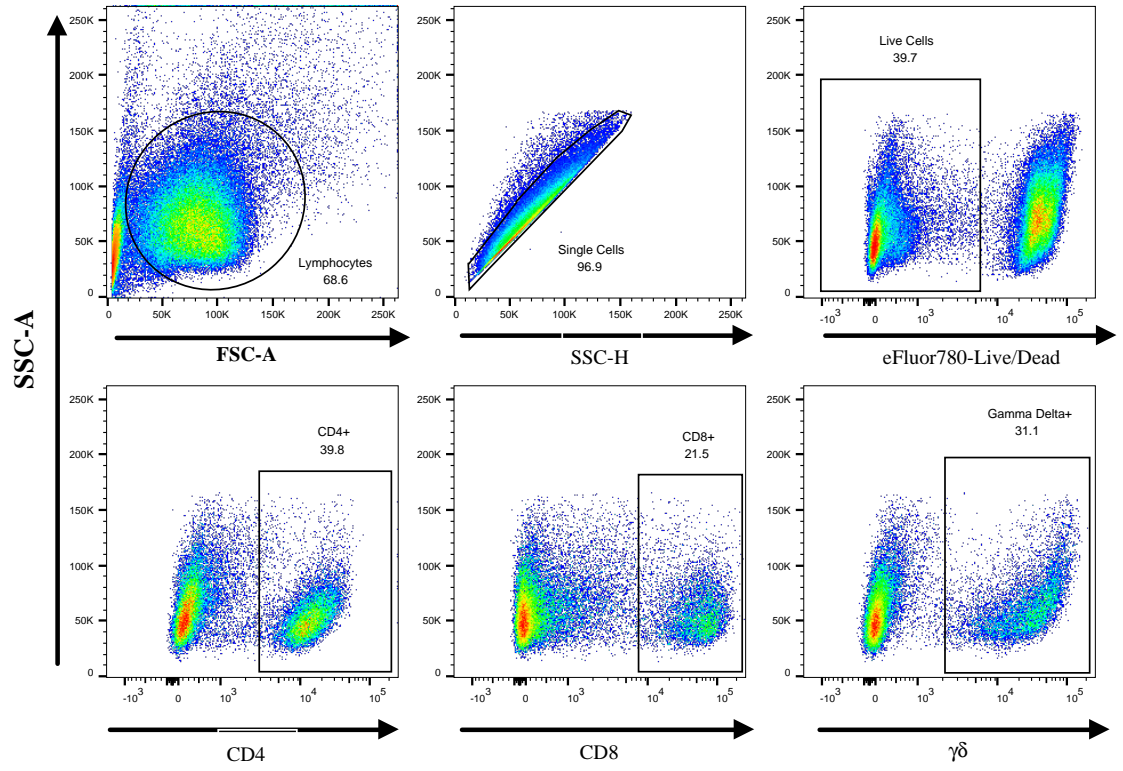

Supplement: Supplementary Figure S2 — Gating strategy for flow cytometry analysis of bison lymphocytes. Representative dot plots show the gating strategy used to define lymphocytes, singlets, live cells, and major T cell subsets (CD4, CD8, γδ). [file Image_2.pdf]

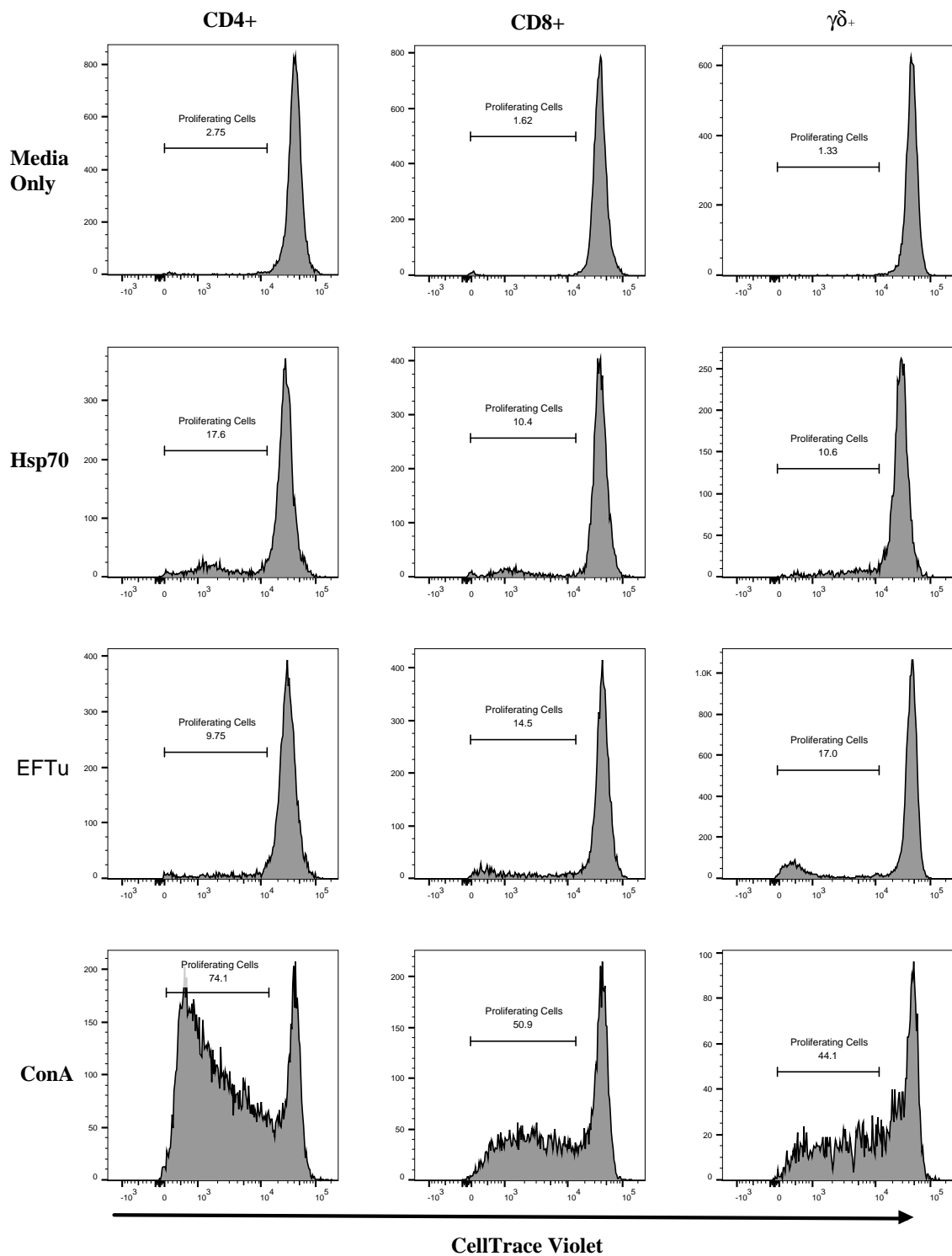

Supplement: Supplementary Figure S3 — Proliferation of bison T cell subsets. Representative histograms showing the CellTrace Violet staining of CD4, CD8, and γδ T cells following culture in media only, Mycoplasma bovis Hsp70 and EFTu, and ConA. [file Image_3.pdf]
